# Exploring experiences of quarantined people during the early phase of COVID-19 outbreak in Southern Nations Nationalities and Peoples’ Region of Ethiopia: A qualitative study

**DOI:** 10.1371/journal.pone.0275248

**Published:** 2022-09-30

**Authors:** Ararso Baru Olani, Nega Degefa, Zeleke Aschalew, Mekdim Kassa, Tesfaye Feleke, Girma Gura, Sarah Namee Wambete

**Affiliations:** 1 College of Medicine and Health Sciences, Arbaminch University, Arbaminch, Ethiopia; 2 Department of Psychology, School of Pedagogical and Behavioral Science, Arbaminch University, Arbaminch, Ethiopia; 3 Save the Mothers East Africa, Uganda Christian University, Mukono, Uganda; University of Auckland, NEW ZEALAND

## Abstract

**Background:**

Ethiopia enforced extremely rigorous contact tracing and mandatory quarantine for all suspected contact and travelers entering the country for a period of 14-days duration during the early phases of the COVID-19 outbreak. Several studies investigated the experience of quarantined people because of COVID-19 or previous outbreaks. However, quarantine is often perceived differently in different cultures because of its historical association with class, gender, ethnicity, politics, and prejudices. To our knowledge, there is limited literature on quarantine experience in Ethiopia related to either COVID-19 or other infectious diseases. Therefore, this study was aimed to explore quarantine experience of people in Southern Nations Nationalities and Peoples’ Region (SNNPR) of Ethiopia during early phase of COVID-19 pandemic.

**Methods:**

The study implemented an exploratory qualitative research design using a phenomenological approach. Face-to-face in-depth interviews were conducted with purposively recruited 29 respondents. Digitally recorded audio files have been listened to several times and verbatim transcriptions were done. The transcribed narratives were examined independently and content analysis was carried out through reading and re-reading the verbatim several times, open coding, grouping, categorizing, and abstracting the final themes.

**Results:**

Three broad themes were identified and characterized the experiences of quarantined people due to COVID-19. These themes were a) handling of the suspected person, b) adverse effects of quarantine and c) coping strategies. In addition, quarantine refusals; injustice in quarantine; quarantine errors; psychological distress; physiological changes; social effects; financial losses; personal and social coping strategies were the emerged sub-themes.

**Conclusions:**

This study explored a range of complex experiences of quarantined people because of the COVID-19 outbreak in SNNPR. The quarantined people included in this study were adversely affected psychologically, physiologically, socially, and economically. They also experienced quarantine errors and injustice. There is a need to gather clear justification for close contact before forcing the suspect for mandatory quarantine. In addition, there is a need to develop risk communication strategy to approach suspected contacts for quarantine. Moreover, assessing psychological, physiological, social, and economic impacts of quarantine on the individuals while they are in quarantine and after release could be important. The use of personal and social coping strategies including psychosocial support may lessen the adverse impacts of the quarantine.

## Background

Quarantine is the restriction of movement of individuals who are not ill, but who may have been exposed to an infectious illness, to prevent the spread of the disease [1]. The primary purpose of quarantine is to prevent the transmission of an infectious agent from those potentially incubating diseases [2].

Quarantines can be applied at the individual, group, or community level, and usually involves restrictions on the home, hotel, or designated facility [3]. It can be voluntary, mandatory, or coercive [4]. In response to the COVID-19 outbreak, Ethiopia enforced extremely rigorous contact tracing and a 14-day mandatory quarantine for all suspected contact during the early phases of the outbreak. In addition, the country enforced a 14-day mandatory quarantine period for all travelers entering the country at their own expense [5].

Although quarantine has risk reduction effects against an emerging epidemic, it is not devoid of adverse effects on quarantined people and their families [6]. These impacts could be followed by either short or long-term consequences [6, 7]. Experience from Severe Acute Respiratory Syndrome (SARS) and Ebola outbreaks evidenced that quarantine is often an unpleasant experience as there are possibilities of separation from loved ones, loss of freedom, boredom, and uncertainty over disease status [8, 9]. Quarantined people may experience acute stress disorder, anxiety, intrusive thoughts, flashbacks, irritability, insomnia, nightmares while in quarantine or after being released from quarantine [6, 10–12]. Quarantined people, their families, and friends could also experience stigma and discrimination due to mandatory contact tracing and quarantine [12]. Moreover, quarantine could pose serious economic distress to quarantined individuals and their families [9].

Several studies have dealt with quarantine experiences and evaluated their emotional, psychological, social, and financial effects during previous outbreaks such as SARS, Middle East Respiratory Syndrome (MERS), and Ebola [2, 9, 13, 14]. Similarly, several studies investigated the experience of quarantined people amid COVID-19 [15, 16]. However, quarantine is often perceived differently in different cultures because of its historical association with class, gender, ethnicity, politics, and prejudices [4]. Studies suggested that quarantine experience could be varied with quarantine policy, access to information, financial and cultural backgrounds [6, 9, 12–14, 17]. The issue of disparity in the impact of COVID-19 across culture, economy, and politics are also striking [18, 19]. To our knowledge, there is a dearth of literature on the experience of quarantined people for COVID-19 or other contagious diseases in Ethiopia. Therefore, this study was aimed at exploring the experiences of quarantined people during the early phase of the COVID-19 outbreak in Ethiopia.

## Methods

### Study design

The study implemented an exploratory qualitative research design based on a phenomenological approach. A qualitative study design was recommended to investigate and describe all phenomena including all kinds of human experience, as the phenomena appear in their fullest breadth and depth [20]. Phenomenology allows exploration of the lived experience of an individual [21, 22]. Therefore, qualitative design using a phenomenological approach was deemed to be the most appropriate for a deeper understanding of the phenomenon under investigation.

### Study setting and period

The study was conducted in Southern Nations, Nationalities, and Peoples’ Region (SNNPR), which is one of the ten regional states that formed the Federal Democratic Republic of Ethiopia. The region borders Kenya to the South, South Sudan to the Southwest and West, Oromia regional state to the North and East, and Gambela regional state to the West. The region is home to over 45 indigenous ethnic groups. SNNPR is the most densely populated region in Ethiopia [23]. Quarantine centers in Gamo zone, Woloyta Zone, Halaba Zone, Hadiya Zone, Silte Zone, Konso Zone, and Hawasa city were purposively selected and included in the study. The study was conducted from June 20 to July 25, 2020.

### Eligibility criteria and population

The eligibility criteria include: individuals aged 18 years or above, regardless of quarantine type, viz; forced, coerced, or voluntary quarantine, and stayed in quarantine center because of the following reason/s: (1) having close contact with a confirmed COVID-19 positive person or (2) having close contact with the suspected person for COVID-19 or (3) having travel history to COVID-19 positive reported area.

### Sampling and data collection procedures

The investigators used a purposive sampling approach. Before conducting the interviews, participants’ information and contact numbers were obtained from quarantine centers’ registries. Participants with different cultural, religious, educational, professional, and economic backgrounds were purposively screened from the registry and contacted through the focal person of each quarantine center. Face-to-face in-depth interviews were conducted with eligible and interested participants under strict physical distancing and other preventive measures. A semi-structured interview guide was prepared in English by reviewing previous works of literature [6, 8–14]. The interviews were held in Amharic and audio recorded. Before conducting the interview, the tool was translated to Amharic guided by WHO’s tool translation guideline [24]. Accordingly, the forward translation of the interview guide was done by ZA and ND, who were members of the research team, health care professionals, and fluent in Amharic. Expert and back translations were also done to meet the aims of the study. The sample required for this study was decided by the concept of theoretical saturation [25]. Based on the suggestion, we explored lived experiences of 29 individuals as determined by the research team. Each interviewed individual was compensated 300 ETB (equivalent to 8.6 USD at the rate of data collection moment). The interviews were recorded for ranging from 22 to 47 minutes.

### Data analysis

Participants were invited to describe their experience of quarantine in detail including the situation before quarantine, experience at the center, and after being released from the center. Digitally recorded audio files have been listened to repeatedly, and verbatim transcriptions were done by AB, ND, and ZA. The transcribed narratives were examined independently by AB, ND, and ZA using inductive thematic content analysis. The analyses were carried out through reading verbatim several times, open coding, grouping, categorizing, and abstracting the final themes [26, 27]. To validate the analyses, multiple coding, peer review, and discussion were made until an agreement was reached [28, 29].

### Ethical consideration

The ethical clearance was obtained from the institutional review board (IRB) of Arba Minch University. A letter of support was written by Arbaminch University to respective institutions. The purposes and importance of the study were explained to the participants of the study and all participants provided written informed consent before the interview. The respondents were informed that participation in the study was voluntary. In addition, the respondent had the right to withdraw from the study at any time during an interview without the urge to explain the reason for withdrawal. The participants were assured that the data will be handled exclusively by the investigators, used only for the study, and no one will be able to recognize them in the report. The confidentiality of information collected from the participants was maintained.

## Results

A total of 29 in-depth interviews were carried out among 18 males and 11 females to explore experiences of quarantined people because of contact suspects for COVID-19 in SNNPR, Ethiopia. The participants’ age ranged from 19 to 65 years. Individuals coming from different occupational backgrounds including farmers, drivers, merchants, daily laborers, students, nurses, doctors, and members of parliament were interviewed and included in the study. The majority of the participants experienced mandatory quarantine (Table 1).

**Table 1:** Characteristics of the study participants.

Characteristics	Frequency (n = 29)	Percentage
**Age in years**		
≤20	3	10.3
21–35	11	37.9
36–50	8	27.6
>50	7	24.1
**Sex**		
Male	18	62.1
Female	11	37.9
**Quarantine type**		
Mandatory	21	72.4
Coerced	3	10.3
Voluntary	5	17.2

The findings from content analyses were categorized into three main themes namely: handling of the suspected person, adverse effects of quarantine, and coping strategies. In addition, the findings were further categorized into the emerged sub-themes viz., the first and second themes each categorized into three and four sub-themes respectively, and, two sub-themes for the third theme. Details on each theme and sub-theme are provided below.

### First theme: Handling of the suspected person

This theme was classified into four sub-themes namely: quarantine refusal, injustice, and quarantine errors.

#### Quarantine refusal

Quarantine refusal was experienced by the majority of the participants including health care providers. Denial, under communication, and lack of preparation for quarantine were reasons provided for violence with legal enforcing bodies.

For example, one of the quarantined people during the early days of the first COVID-19 report in Ethiopia lamented as follows:

*"There was no ground base to force me for quarantine*. *My close contact already tested negative*.*"* ~ A young medical doctor.

Contact denial was identified as the reason for quarantine refusal by several participants. For example,

*"I cannot understand why you (the police officers) want to spoil me*. *I am a healthy person*. *I do not have any illness*. *I am telling you that I cannot follow you to the quarantine center*. *You can gun down me here by the bullet"* ~A merchant woman whose work partner tested positive for COVID-19.

Under preparation for quarantine was another reason for quarantine refusal. For example,

*“I am not prepared at all*. *You are forcing all of my family members including young children*. *Who will be left at home and looking after my properties*? *You are saying leave the house to be looted by robberies and the cattle for the hyenas*.*”* ~65 years old farmer

Lack of communication or under communication was a frequent experience during the early days of the COVID-19 outbreak according to the study participants. For example, one of the study participants said:

*"The law enforcing bodies knocked on my house and ordered me to follow them without any further explanations*. *I argued with them and refused the order although there was no further action I can consider”*~ A woman in her late thirties

#### Injustice

The study participants noted that disrespect, intimidation, harsh treatment, and detention were their frequent experiences related to quarantine.

Several respondents blamed legal enforcement bodies for disrespect and harsh treatment. The following quotes were some of the examples given by the study participants:

*“The way the police officers forced me was similar to the situation we were experienced during the ‘dergue’ regime (a military government that ruled Ethiopia from 1974 to 1991)*. *They (the police officer) use all available forces to take me to the quarantine center*.*”* ~53 years old farmer

*"About ten police officers with a gun arrived at my house*. *I was scared and felt guilty because of the numbers and activities of police officers seemed I committed the worst crime such as homicide"* ~35 years old woman

Intimidation by law enforcement bodies was reported by the majority of respondents who were forced into quarantine. One of the respondents narrated the situation as follows:

*"…I was traveling back home from University following the announcement of lockdown*. *At the gate to Hawasa city*, *police officers with guns loudly shout at us saying to get out of the car*. *Do not try to escape*. *If you do so*, *you will be hit by the bullet*. *I was shocked as I did not have information on what happened to us at the moment*. *Later they took us to the quarantine center and they told us someone escaped from Addis Ababa to Hawasa following tested positive using public transport and they were looking for the person”* ~19 years old boy who was a first-year university student

Some of the participants were also detained at the rural police stations for days before reaching a nearby quarantine center. The way they were treated at police stations was similar to that of people sentenced for conducting crime according to their expression. One of the respondents narrated the situation as follows:

*“I was detained in rural prison like a person suspected for crime in rural prison for two days before they transferred me to quarantine center”* a 31 years old man.

#### Quarantine errors

Some respondents blamed the authorities for the quarantine mistake due to failure to confirm the identity of the suspect that need quarantine. In some cases, individuals were wrongly quarantined because of a wrong report to quarantine centers by the individuals who were in personal conflicts with the suspects. For example one of the respondents described her experience as follows:

*" I had a personal conflict with one of my neighbors*. *She holds a grudge and reported me to the task force (which was established following the COVID-19 outbreak in Ethiopia)*. *I had no either travel history or close contact with suspected person or any clinical manifestations”*~ 37 years old woman

### Second theme: Adverse effects of quarantine

**Fig 1:**
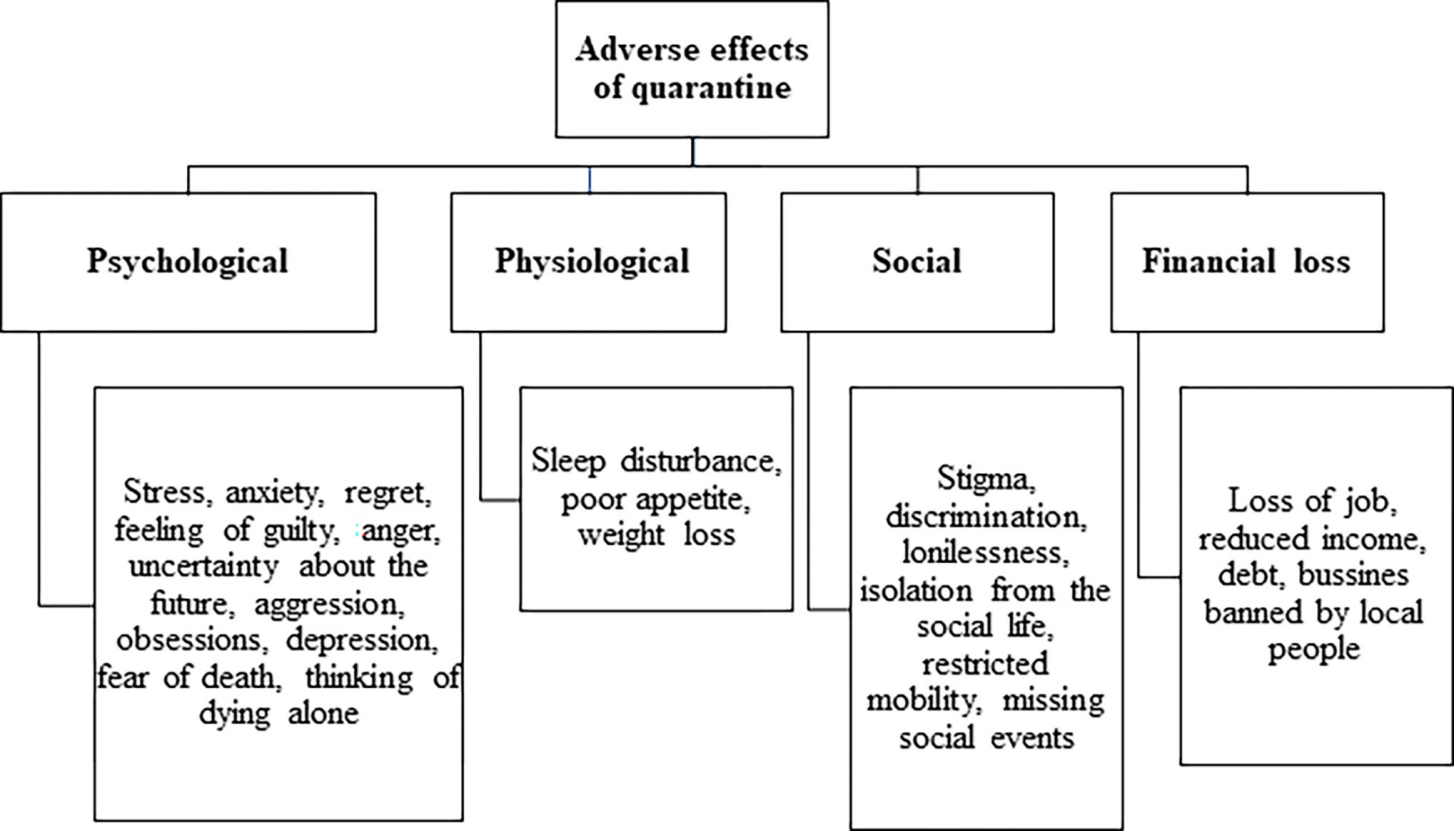
Adverse effects of quarantine.

Four major themes that described the adverse effects of quarantines were identified in this study. These are psychological distress, physiological changes, social effects, and financial losses (Fig 1).

#### Psychological distress

The causes of psychological distress were related to anxiety, feeling of guilt, confusion, anger, uncertainty about the future, aggression, obsessions, depression, fear of death, fear of death without mourning, fear of contracting the disease from the center, and suicide ideation were identified psychological and mental health effects of quarantine in this study. The stressors were varying from person to person, however, they had common predisposing causes in most of the cases which were fear of the virus.

Uncertainty about the disease led some of the respondents to phycological distress such as anxiety and fear of death. A young medical doctor said that,

*“…I was too scared because it is my turn to help my parents*. *They invested their money and time in me*. *Rather than being a newly graduated young doctor*, *I haven’t achieved anything so far*. *If I tested positive what would be my fate*? *I could die young*. *A death before accomplishing the mission*. *What would be my family’s reactions*?*"*

Uncertainty about the disease also caused psychological distress to some respondents who came from a cultural background where property inheritance to children has cultural value and personal satisfaction. For example, one of the respondents said,

*"I heard that the disease is a killer*. *I feared that if the entire family and I could die of the virus*, *who would inherit my properties*? *Am I be the last person of my clan without even having a person going to inherit me*?*"*~ 46 years old farmer

Fear of infection from the center was another concept that causes psychological distress to quarantined people. A 26 years old man said,

*"My stay at the center was quite boring*. *I shared a single room with six suspected men*. *Although I was confident regarding my status*, *I was too stressed because the chance of contracting the disease from another suspected person at the center was tremendous*.*”*

Another concept that caused psychological distress was interpersonal conflicts at the quarantine centers. For example one of the respondents said,

*"…My stay in quarantine was full of depression*. *I was asked to report my close contacts*. *I mentioned several people including my boss*. *She was a merchant at the local market in the area and I used to push a wheelbarrow for her*. *The police officers brought the woman to the center*. *She yelled at me*, *abused me verbally*, *and even stoned me*. *I was very irritated the day she cursed my newly born son saying ’let God kill the newborn*!*’ I preferred staying in the room given to me than going outside fearing her verbal abuses*.*” ~*a 23-year-old man

Interpersonal conflicts were also reported between quarantined people and the authorities. For example, some of the participants reported that they conflicted with health care providers and law enforcement bodies for no justifiable reasons, which later ended with regret. One of the respondents lamented as follows:

*"…Unjustifiable disputes with the health care workers and security guards were my frequent experience at the quarantine center*. *I yelled at health care providers several times and regretted”~* a mother of two and merchant

The other cause of psychological distress was being in quarantine with my children. For example, one of the respondents lamented the situation as follows:

*“I was quarantined with my three children and all of them were less than 10 years old*. *They want to go outside but they were not allowed to do so*. *They never stayed in a little room for the whole day*. *Imagine a child coming from a rural area and being forced to stay in a little room*. *Sometimes my children cry for hours*. *The worst story was that my children were repeatedly asked for food that I cannot provide for them*. *They were repeatedly asking for food saying*, *‘Dad I need food*, *I am hungry…crying’*. *I do not want to remember those bad days (crying)*.*"*

#### Physiological changes

Sleep disturbance, poor appetite, and weight loss were reported by the majority of the study participants. Fake news, uncertainty about the disease, anxiety, a new environment, and less preparation in the quarantine centers were identified factors that led quarantined people to physiological changes.

For example, the following narratives showed how fake news led to physiological changes at the quarantine center:

*"I heard a bad story about how a dead body from COVID-19 was handled by one of the quarantine people*. *He said that the dead body is not allowed for burial*, *instead cremation (Ethiopians do not cremate dead bodies)*. *Starting that night*, *I started to ask myself such questions*. *Am I going to be burned like wood*? *how my community is not allowed to bury me*? *Following the news*, *I had poor appetite*, *difficulty in falling asleep*, *and nightmares until I was informed tested negative" ~ 45 years old man*.

Some respondents said that their weight loss was attributable to shortage and lack of food varieties at the center in addition to stress about the disease.

For example, a 65 years old man described his experience as follows:

*"Food inadequacy at the center was a great challenge to me*. *The food they serve to me was not even sufficient to feed children*.*"*

Another man lamented his experience as follows:

*"There was no food choice at the center*. *For breakfast*, *rice with bread was the only food provided by the center*. *We usually repeat the same food for lunch and dinner*. *Unfortunately*, *It was my first time eating rice and I was unable to adapt to eating rice*. *I asked the authority either to provide alternative food or allow me to buy food from outside*. *I was not allowed either*.*"*~46 years old farmer

#### Social effects

Experience of discrimination, stigma, and loneliness was majorly reported as social effects of quarantine in this study. Other minor mentions were isolation from social life and missing social events. Stigma and discriminations were common experiences to quarantined people both at quarantine centers and after being released from the centers.

One of the respondents lamented his experience at the center saying that,

*“It was Ramadan month and I was on fasting the whole day*. *The ‘iftar’ time was up but the food was not served to me on time*. *I asked the security officers to buy food for me*. *However*, *he responded to me with an unexpected answer saying ’your money is contaminated*, *therefore*, *I cannot collect from you*.*’ I was nervous and envy death at the moment"* ~A 45 years old man

Perceived discrimination at the center was another experience reported by some of the participants. For example,

*“Food is served on a plate according to my culture*. *However*, *what I experienced at the quarantine center was odd to me*. *They served me with a thin plastic bag*, *which was disrespectful and discriminatory to me*. *Older people are highly respected in my community*.*”* ~65 years old man.

After being released from quarantine centers some respondents nicknamed COVID-19. Such experiences were mainly reported by individuals living in urban areas. For example,

*"Some people are calling me with the nickname ’COVID-19’ in public"* ~ A daily laborer and young man.

In some cases, the discrimination was extended to the family of quarantined people. For example,

*“…My family members were prevented from entering the market by local militia following a loud shout from people in the market*.*”* ~ A 53 years old farmer.

Loneliness was a common experience for several respondents at the quarantine center. Some respondents described quarantine as detachment from socialization. One of the respondents described his quarantine experience as follows:

“*Bismillah…prison is a better place*. *In prison*, *you get imprisoned with a human being and you share the room with people*. *However*, *in quarantine*, *I am forced to sleep alone in a single room*. *At the prison I used to talk to other people physically*, *in the quarantine center I was allowed to talk and play physically with others”* 42 years old man.

#### Financial losses

The financial loss associated with quarantine in this study was largely due to the restriction of the movement. However, some of the respondents blamed the sudden and forced quarantine of entire family members for their losses.

For example, one of the respondents described the situation that led her to financial loss and debt as follows:

*“I am ensete (staple food in the area) products*, *retailer*. *No one was left at home as entire of my family members were forced into quarantine*. *After two week stay at the center*, *I returned home but the ensete estimated to be 20*,*000 birrs (equivalent to 600 USD) was already spoiled*. *I am in debt at this moment to support my family”* ~ A 46 years old retailer woman

Another respondent said:

*"I am a farmer and we wait a season to plow the land*. *I lagged to farmland*. *That two weeks were crucial to me*. *I left without seeding maize*, *millet*, *and sorghum on time*.*"* ~ A 53-year-old farmer

In some situations, respondents there were respondents whose income was reduced after being released from quarantine due to discrimination from the community. For example, one of the respondents lamented his experience as follows:

*"I have a flour milling machine to run business*. *Most of my customers banned the house hearing that I was in quarantine suspected of COVID-19*. *Since then*, *my income is reduced more than by half”* ~A 48 years old man.

Some individuals reported their contacts and lost their job. The followings are examples taken from the interview:

*“*…*I was avenged by my boss just for reporting as I had close contact with her*. *She fired me from the work and currently I am jobless (crying)”* ~A 23 years old man.

*"I used to support my daily needs by riding a motorbike*. *However*, *after the quarantine*, *I lost my job because the owner of the motorbike was forced to quarantine after I reported him as one of my close contacts*.*"* A 25-year-old man.

**Schematic representation of adverse effects of quarantine.** Fig 1.

### Third theme: Coping strategies

Both personal and social supports were identified as coping strategies among quarantined people in the present study.

#### Personal supports

Strengthening of spiritual attachment with God or Allah has majorly mentioned coping strategy with quarantine in this study. Praying for God’s mercy and salvation, and strengthening personal bonding with God were reported by most of the respondents. In addition, listening to spiritual music and reading spiritual scripts were minor mentioned by the respondents. The followings were practices of spirituality described by the respondents:

*“Praying helped me a lot during my stay in the quarantine*. *I frequently prayed for God’s salvation*, *for the sin I did both intentionally and unintentionally*.*”* ~A 35 years old woman

*"God was the only hope I rely on at the center*. *Truly speaking*, *quarantine further strengthens my relationship with God*.*"* ~28 years old man

*“…It is a disease*, *it came from Allah*. *Consistent praying for the Mercy of Allah was the sole action I used to do at the center to cope with the encountered crisis*.*”~* 47 years old man

Another personal support identified in this study was problem-oriented coping strategies. The commonly mentioned strategies were reading books, taking shower, physical exercise, refraining from daily updates checking on news regarding COVID-19, preventing themselves from disclosing their quarantine stay. Browsing the internet, using social media, and phone calls with friends and family were also mentioned by some individuals particularly health care workers as coping strategies at a quarantine center. The followings are examples of quotes selected from the participants’ experiences:

*“Listening to music*, *taking shower*, *and doing some kinds of physical exercises at the quarantine center was my frequent experience to forget about COVID-19" ~* a 19 years old female

*“I can say that accessing technology such as internet and phone reduced my stress at quarantine center”* ~ A young medical doctor

#### Psycho-social supports

Social support from friends and family, and psycho-social support from health care workers and volunteers helped most of the quarantined people during their stay at the centers. The respondents received psychosocial support both in formal and informal ways although the degree of support varies across respondents. For example, compared to non-healthcare providers, quarantined healthcare providers received less psychological support either from volunteers or healthcare providers. However, quarantined healthcare workers received more social support including access to the use of technologies, food services with varieties, and access to visitors maintaining physical distancing compared to non-healthcare providers in this study. The experiences of some participants are lamented as follows:

*“My first few days at the center were normal*. *I had access to the internet*, *mobile phone*, *and other essential pieces of stuff*. *Later*, *I experienced ignoring daily habitual actions such as brushing teeth*, *washing faces*, *and others*. *Friends of mine and co-workers noticed the situation and provided me some kind of psychological despite it being informal*. *My quarantine stay could have been worst without those encouragements and motivational speeches*. *I can witness that health care workers barely receive psychological support although they are at high risk to experience phycological distress with similar severity non-healthcare providers faces because of COVID-19 related quarantine”* ~26 years old Medical doctor

*“The first three days at the quarantine center were quite difficult as I had the habit of smoking*. *I received psychological support at quarantine including some counseling related to COVID-19 and smoking*. *It helped me to cope with quarantine and even reaching on a decision to stop cigarette smoking*. *I didn’t smoke during the fourteen days stay in quarantine and after release as well”* 26 years old man

## Discussions

The main goal of this study was to explore the experiences of quarantined people due to the COVID-19 Pandemic in SNNPR, Ethiopia. Three broad themes were identified that characterized the experiences of quarantined people due to COVID-19. These themes are a) Handling of the suspected person, b) adverse effects of quarantine, and c) coping strategies.

Our findings revealed that there was mishandling of participants suspected to be infected with COVID-19. Some participants reported denial of and unpreparedness for being quarantined. Similar findings were reported in a qualitative study done in Uganda on people in institutional quarantine [30]. Additionally, poor communication, lack of transparency about the virus, and how to conduct themselves during the quarantine were experienced. Some participants were not given any information as to why they were being quarantined and others were given limited information which brought about a lot of confusion [30, 31]. Another study was done in Kashmir also reported feelings of shock and denial about being quarantined among participants [32].

The participants also experienced police brutality in this study. A couple of African studies have shown that police, military, and law enforcement officers use intimidation, threats, and even imprisonment to strictly enforce COVID-19 quarantine and lockdown measures [33, 34]. The possible reason for police brutality is probably because of the belief that instilling fear in people suspected to be infected with COVID-19 will enable compliance with quarantine measures [16, 35]. However, brutality by law enforcement officers has been shown to cause more harm than good [36] and additionally, adherence to the quarantine measures mainly stems from inadequate or complete lack of information on COVID-19. The relationship between lack of knowledge of COVID-19 and poor adherence to quarantine measures has been reported in some studies [37, 38].

A previous study conducted in Uganda reported that people were mistaken to be having COVID-19. Their neighbors called the police to investigate them, therefore leading to unnecessary quarantine because they did not have any contact with an infected person [39]. This study also found that one participant’s hateful neighbor called the police to her home to forcefully quarantine her even though she had no travel history or contact with an infected person.

The findings of this study revealed that quarantine stations or centers were underprepared. Participants reported a shortage of food, water, and sanitary materials in quarantine centers, which made it difficult for them to withstand their time at the quarantine centers. Other studies reported that food and basic supply shortage at the quarantine centers caused frustration and anxiety among the participants [30, 37, 38].

This study also found that participants experienced several adverse effects of being under quarantine for COVID-19. The quarantined people in this study experienced adverse mental health outcomes such as anger, stress, anxiety, feeling of guilt, confusion, aggression obsessions, and depression. The impact of COVID-19 on anxiety, anger, and depression was previously investigated and reported comparable results [40–44]. Additionally, participants also had fear of death especially without mourning guaranteed, fear of infecting their families, the uncertainty of the future, and fear of contracting the disease from the quarantine center when they were confident they did not have the disease officially [45–47].

Physiological effects such as insomnia, loss of appetite, and weight loss also contributed to negative experiences at the quarantine center. This mainly stemmed from the bad stories and news they heard about COVID-19 [48, 49]. Social effects which included stigma, discrimination, and loneliness were reported by the participants. Several studies have also reported stigma and discrimination against quarantined participants [50–52]. In this study, participants also faced economic effects such as loss of jobs, reduced income, and inability to support families as a result of financial failure due to quarantine restrictions. Negative economic effects as a result of quarantine restrictions due to the COVID-19 pandemic have been reported by other studies [53–55].

Quarantined participants resorted to coping strategies to get through their time at the quarantine center; participants resorted to personal coping strategies such as spirituality and problem-oriented coping strategies. This study found that participants prayed and meditated more to cope with the quarantine situation. Similarly, a study done in the USA among college students reported that the participants resorted to meditation and spirituality to cope with the quarantine stress [56]. However, another study done across South Asian countries reported a decline in religious beliefs during quarantine [57]. Social support such as friends, family, and health workers also helped participants to cope with the quarantine stress. A study done in Ethiopia reported that quarantined participants who had good social support were less likely to experience anxiety during quarantine [58].

### Limitations of the study

The current study has some limitations. The recruitment of the participants for the study was entirely from SNNPR. Although this study involved participants from different cultures and a wide geographical environment, the information used in this study are still does not representing entire parts of Ethiopia. Therefore, the generalization of these findings should be with caution as living environment and cultural differences could affect the quarantine experience. As the study was entirely qualitative, the study lacks quantifiable measurements. A quantitative study is suggested for future studies for a better understanding of the adverse effects of quarantine.

## Conclusions

This study explored a range of complex experiences of peoples quarantined for COVID-19 in SNNPR. The study identified experiences related to the handling of the suspected person including quarantine refusal; injustice in quarantine such as disrespect, intimidation, harsh treatment, and detention; and quarantine errors were identified as major experiences. In addition, adverse effects of quarantine include psychological distress such as anxiety, feeling of guilt, confusion, anger, uncertainty about the future, and aggression; physiological changes such as reduced appetite and sleep disturbance; social effects such as stigma, discrimination, and loneliness; and financial losses such as reduced income, debt, and loss of job were identified. Moreover, coping strategies with quarantine including personal and social support were identified.

This study explored a range of complex experiences of peoples quarantined for COVID-19 in SNNPR. The quarantined people included in this study were adversely affected psychologically, physiologically, socially, and economically. They also experienced quarantine errors and injustice.

## Implications

The present study findings indicate the need to gather clear justification on the suspected individual for close contact before forcing for mandatory quarantine. In addition, the findings indicate the need to develop a strategy for risk communication in approaching individuals for quarantine. There is also a need to assess the psychological, physiological, social, and economic impacts of quarantine on the individuals while they are in quarantine and after release. Furthermore, strengthening personal and social coping strategies including psychosocial support may lessen the adverse impacts of the quarantine. The current findings are useful for policymakers in Ethiopia and beyond as input in responding to evolving and emerging communicable diseases.

## Supporting information

S1 File Interview guide. (DOCX)

## Abbreviations

ABO: Ararso Baru Olani
ETB: Ethiopian Birr
GG: Girma Gura
MERS: Middle East Respiratory Syndrome
MK: Mekdim Kassa
ND: Nega Degefa
SARS: Severe Acute Respiratory Syndrome
SNNPR: Southern nations, nationalities and peoples’ region
SNW: Sarah Namee Wambete
TF: Tsfaye Feleke
WHO: World health organization
ZA: Zeleke Aschalew
