# Supplementary material for: Exploring experiences of quarantined people during the early phase of COVID-19 outbreak in Southern Nations Nationalities and Peoples’ Region of Ethiopia: A qualitative study
Source: PLoS One. 2022 Sep 30;17(9):e0275248. doi: 10.1371/journal.pone.0275248 (PMC9524644; doi:10.1371/journal.pone.0275248)
Supplement: S1 File — (DOCX) [file pone.0275248.s001.docx]

**INTERVIEW GUIDE FOR QUARANTINE EXPERIENCE STUDY**

| **IDENTIFICATION** | |
| --- | --- |
| LOCALITY NAME ___________________________________________የመኖሪያ ስፍራ _________________________  AGE­­­­­­­­­­­­­­­ __________እድሜ  SEX _______ጾታ  MARITAL STATUS ________የጋብቻ ሁኔታ  OCCUPATION _____የስራ አይነት  EDUCATIONAL LEVEL ________ የትምህርት ደረጃ _______________  DATE OF ADMISSION TO QUARANTINE CENTER _____________________ወደ ለይቶ ማቆያ የገቡበት ቀን ________________  DATE OF DISCHARGE FROM QUARANTINE CENTER __________________ከ ለይቶ ማቆያ የወጡበት ቀን_____________  HISTORY OF CONTACT WITH COVID-19 PATIENT ____________________ ከኮቪድ 19 በሽተኛ ጋር የንክኪ ታሪክ ________________  HISTORY OF FLIGHT TO OR FROM RISKY AREA ______________________የጉዞ ታሪክ ______________________  ARE YOU WILLING TO PARTICIPATE? YES NO  yes  በቃለ መጠይቁ ላይ ለመሳተፍ ያሎት ፍቃደኝነት? አዎ አይደለዉም  QUARANTINE TYPE : VOLUNTARY. MANDATORY. COERCED | |
| ***INTERVIEWERS PROFILE***  **_____________________________NAME**  **______________ DATE**  **______________TIME OF INTERVIEW** | |
| ***EXPERIENCE BEFORE QUARENTINE*** | |
| HOW DO YOU COMPLY WITH PUBLIC HEALTH MEASURES OF COVID-19 PREVENTION?  የ ኮቪድ 19ን ወረርሽኝ ለመከላከል የሚወሰዱ መሃበራዊ እርመጃዎችን ይተገብራሉ? | *Probe: (such as hand washing, use of facemask, avoid crowd or public, avoid hand shaking, physical distancing (Minimum of 1.5 M), use alcohol-based hand sanitizer)* |
| WHAT DO YOU THINK ABOUT THE REALITY OF COVID-19 BEFORE QUARENTINE?  በውነቱ የ ኮቪድ 19 በሽታ አለ ብለው ያስባሉ? | *(Probe if there is denial of COVID-19)* |
| HOW DID YOU EXPLAIN THE HANDLING OF THE GOVERNMENT AUTHORITY OR HEALTH CARE PROVIDER WHILE THEY TOOK YOU TO THE QUARENTINE CENTER?  የመንግስት አካላት ወይም የ ጤና ባለሞያው ወደ ግዚያዊ ለይቶ ማቆያ በምትገቡ ግዜ ያላችው ክብር ፣ ርህራሄ እና መስተንግዶ እንዴት ነበር? | *Probe (Showed compassion, respect and dignity)* |
| ***EXPERIENCE DURING QUARENTINE*** | |
| HAVE YOU GET ANY INFORMATION ABOUT QUARENTINE, BEFORE YOU ENTER THE QUARENTINE CENTER?  ወደ ኳረንቲን ማዕከል ከመግባቶ በፊት ስለ ኳረንቲን ማንኛውንም መረጃ አግኝተዋል | *Probe (Quarantine protects (self, household, community), compliant because they want to be good citizen or consider it as civic duty, legal reason for compliance, adhered to quarantine because they think of healthy of their loved one)* |
| HOW DO YOU EVALUATE THE MESSAGE GIVEN? | *Probe (reason for being quarantined)* |
| HOW WAS YOUR STAY IN QUARENTINE CENTER?  በ ኳረንቲን ማዕከል ውስጥ የነበሮት ቆይታ እንዴት ነበር? | *Probe: Boredom and frustration, fear of infection, annoyance, worries, loneliness and in adequate supply* |
| WHAT WAS THE REASON FOR THE PROBLEM?  የችግሩ ምክንያት ምን ነበር? | *Probe: Confinement, loss of usual routine, and reduced social and physical contact with others were frequently shown to cause boredom, frustration, and a sense of isolation from the rest of the world* |
| ***POST QUARENTINE EXPERIENCE*** | |
| WHAT DO YOU EXPERIENCE AFTER BEING DISCHARGED FROM QUARANTINE?  ከለይቶ ማቆያ ከተመለስክ በኋላ ምንገጥመህ? | *Probe: Inters of income, job, and reaction of employer’s* |
| HOW WAS THE COMMUNITIES RESPONSE ON YOUR RETURN FROM QUARANTINE CENTER?  ከለይቶ ማቆያ ከተመለስክ በኋላ የማህበረሰቡ አቀባበል ምን ዪመስል ነበር? | *Probe: Avoiding, withdrawing social invitations, being treated with fear and suspicion, and making critical comments* |
